# Critical region in 2q31.2q32.3 deletion syndrome: Report of two phenotypically distinct patients, one with an additional deletion in Alagille syndrome region

**DOI:** 10.1186/1755-8166-5-25

**Published:** 2012-05-02

**Authors:** Susana Isabel Ferreira, Eunice Matoso, Margarida Venâncio, Jorge Saraiva, Joana B Melo, Isabel Marques Carreira

**Affiliations:** 1 Laboratório de Citogenética e Genómica - Faculty of Medicine, University of Coimbra, Coimbra, Portugal; 2 Serviço de Genética Médica, Hospital Pediátrico Carmona da Mota, Centro Hospitalar e Universitário de Coimbra and Faculty of Medicine, University of Coimbra, Coimbra, Portugal; 3 CIMAGO – Centro de Investigação em Meio Ambiente, Genética e Oncobiologia, Faculty of Medicine, University of Coimbra, Coimbra, Portugal

**Keywords:** 2q31.2q32.3 deletion, Critical region, Alagille syndrome

## Abstract

**Background:**

Standard cytogenetic analysis has revealed to date more than 30 reported cases presenting interstitial deletions involving region 2q31-q32, but with poorly defined breakpoints. After the postulation of 2q31.2q32.3 deletion as a clinically recognizable disorder, more patients were reported with a critical region proposed and candidate genes pointed out.

**Results:**

We report two female patients with *de novo* chromosome 2 cytogenetically visible deletions, one of them with an additional *de novo* deletion in chromosome 20p12.2p12.3. Patient I presents a 16.8 Mb deletion in 2q31.2q32.3 while patient II presents a smaller deletion of 7 Mb in 2q32.1q32.3, entirely contained within patient I deleted region, and a second 4 Mb deletion in Alagille syndrome region. Patient I clearly manifests symptoms associated with the 2q31.2q32.3 deletion syndrome, like the muscular phenotype and behavioral problems, while patient II phenotype is compatible with the 20p12 deletion since she manifests problems at the cardiac level, without significant dysmorphisms and an apparently normal psychomotor development.

**Conclusions:**

Whereas Alagille syndrome is a well characterized condition mainly caused by haploinsufficiency of *JAG1* gene, with manifestations that can range from slight clinical findings to major symptoms in different domains, the 2q31.2q32.3 deletion syndrome is still being delineated. The occurrence of both imbalances in reported patient II would be expected to cause a more severe phenotype compared to the individual phenotype associated with each imbalance, which is not the case, since there are no manifestations due to the 2q32 deletion. This, together with the fact that patient I deleted region overlaps previously reported cases and patient II deletion is outside this common region, reinforces the existence of a critical region in 2q31.3q32.1, between 181 to 185 Mb, responsible for the clinical phenotype.

## Background

Standard cytogenetic analysis has revealed to date more than 30 reported cases presenting interstitial deletions involving region 2q31-q32, but with poorly defined breakpoints [1]. After the reports of Van Buggenhout *et al*. [2] and Mencarelli *et al*. [3], Prontera *et al*. [4] was the first to postulate 2q31.2q32.3 deletion as a clinically recognizable disorder. Rifai *et al*. [1] and Cocchella *et al*. [5] reported two additional patients, with the last one refining a critical region for the syndrome and pointing out candidate genes to explain the phenotype. The common clinical features include pre- and postnatal growth retardation, severe mental retardation, distinct facial dysmorphisms, thin and sparse hair, micrognathia, cleft or high palate, relative macroglossia, dacryocystitis (inflammation and infection of the tear sac), persistent feeding difficulties, inguinal hernia and broad-based gait [1].

An already established condition is Alagille syndrome (AGS - OMIM 118450), a multi-system, dominantly inherited developmental disorder [6]. AGS maps to 20p12 and is mainly caused by haploinsufficiency of the Jagged-1 gene (*JAG1-* OMIM 601920), due to mutations in 70% of the cases and to deletions in 3-7% of the patients [6]. The clinical manifestations of the syndrome are highly variable, ranging from slight clinical findings to major symptoms in 5 domains: cardiac, skeletal, ocular, facial and liver [6].

We report two different female patients with chromosome 2 deletions, both *de novo*. Patient I presents a 16.8 Mb deletion in 2q31.2q32.3 while patient II presents a smaller deletion of 7 Mb in 2q32.1q32.3, entirely contained within patient I deleted region. Besides this deletion patient II also presents a *de novo* 4 Mb deletion in 20p12.2p12.3 Alagille syndrome region. Patient I manifests symptoms clearly associated with the 2q31.2q32.3 deletion syndrome, while patient II manifestations are exclusively associated with AGS. As the region deleted exclusively in patient I, and normal in patient II, overlaps the previously reported deletions [3-5], we intent to contribute to the refinement of the critical region responsible for the 2q31.2q32.3 deletion syndrome.

### Clinical report

#### Patient I

Girl with 8 years and 9 months old, the second female child of healthy unrelated parents with an unremarkable family history. The pregnancy was uneventful, without any signs of intrauterine growth restriction and the birth was normal at 36 weeks gestation. Birth weight was 2470 g (50th centile), birth length 44 cm (10th centile) and birth head circumference 32.5 cm (50th centile). At the age of 8 years her height was 124.5 cm (25^th^ centile), her weight 25.3 kg (50^th^ centile) and head circumference 52.5 cm (75^th^ centile). In the first year of life she had recurrent episodes of dacryocystitis and feeding problems, but nowadays she has uncontrollable eating habits which her parents cope with successfully. She has behavioral problems, with some aggressively and an unpredictable humor, but has an active speech. According to the Wechsler Intelligence Scale for Children (WISC), she has mild mental retardation with a full scale IQ score of 69, verbal IQ score of 73 and performance IQ score of 75. Distinguishing features include thick and coarse hair, dry skin, brachycephaly with a large but narrow forehead, deep set eyes and midface hypoplasia. The palate is high and narrow, similarly to her mother and other familiars. She has bilateral tapering fingers, sandal gap and limb muscle hypertrophy with difficulties in motor coordination and fine motor skills.

#### Patient II

Three year-old girl, the third child of a healthy unrelated couple, with an unremarkable family history. She was born at 36 weeks of gestation, but at 34 weeks was identified an oligohydramnios. Cytogenetic prenatal diagnosis due to advanced maternal age was performed in another laboratory and revealed a normal karyotype. At delivery her birth weight was 2164 g (between the 25^th^ and the 75^th^ percentile), birth length 43 cm (3^rd^ percentile) and birth occipitofrontal circumference (OFC) 30 cm (below the 5^th^ percentile). Her Apgar score (9 at the first minute and 10 at the fifth minute) was normal. In the neonatal period she was admitted in a neonatal intensive care unit for 20 days due to a urinary tract infection. Complementary diagnostic exams revealed a congenital heart disease characterized by a subaortic ventricular septal defect, pulmonary stenosis and a patent *ductus arteriousus***.** The renal ultrasound made the diagnosis of a vesicoureteral reflux. Clinical examination at 19 months revealed no significant dysmorphisms with an apparently normal psychomotor development. Her standing height (77.5 cm, 10^th^ percentile) and OFC (46 cm, 25^th^ percentile) were normal, but her weight was below the 5^th^ percentile (9.040 g).

Standard cytogenetic analysis of peripheral blood lymphocytes was performed on both patients revealing imbalances on chromosome 2 that were further characterized by oligoarray-CGH.

## Results

**Figure 1:**
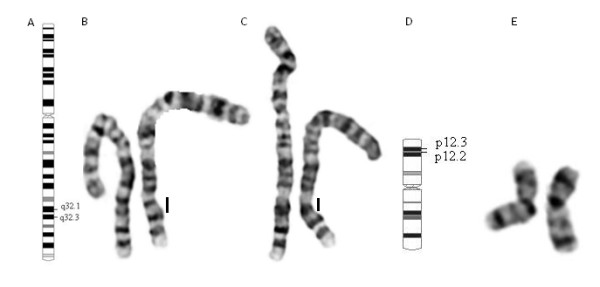
**Conventional cytogenetic results:** Ideogram of chromosome 2 locating the deletion **(A)**, conventional GTG banded pair of chromosomes 2 from patients I **(B)** and II with the deleted chromosome on the right **(C),** ideogram of chromosome 20 locating the deletion **(D)** and pair of chromosomes 20 from patient II **(E).**

Chromosomal analysis of G-banded metaphases from probands and their progenitors showed both patients to have *de novo* (paternity and maternity confirmed) interstitial deletions in chromosome 2 between bands 2q32.1q32.3 (Figure 1A-C). Karyotypes of both girls were 46,XX,del(2)(q32.1 ~ q32.3)dn.

**Figure 2:**
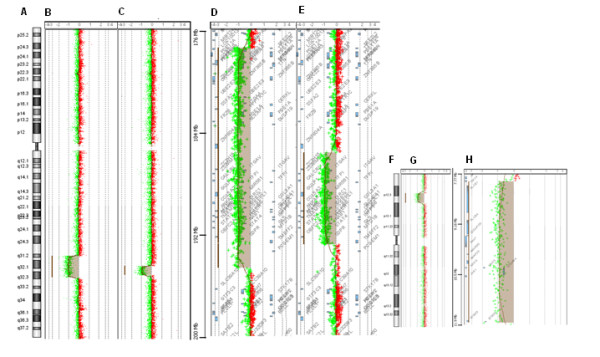
**Oligoarray-CGH ratio profiles for chromosomes 2 and 20:** chromosome 2 ideogram (**A**), Log2 ratio of chromosome 2 probes plotted as a function of chromosomal position for patients I (**B**) and II (**C**), genes involved in the 2q32deletion of patients I (**D**) and II (**E**); chromosome 20 ideogram (**F**), Log2 ratio of chromosome 20 probes plotted as a function of chromosomal position for patient II (**G**), genes involved in the 20p12 deletion of patient II (**H**). Each spot represents a probe, with the patient DNA labeled in red (Cy5) and the control DNA labeled in green (Cy3). Log2 ratios of zero represent equal fluorescence between both samples and the log2 ratio of -1 means copy number loss of the sample DNA.

Oligoarray-CGH analysis confirmed both deletions spanning in patient I about 16.8 Mb of genomic DNA and in patient II 7 Mb (Figure 2A-C). In patient I the deletion involves 72 genes from position 178,121,127 bp (clone A_16_P00537150) to 194,943,948 bp (clone_A16_P36015481), with the proximal breakpoint between 178,112,041 bp and 178,112,127 bp and the distal breakpoint between 194,943,948 bp and 194,976,996 bp (mapped according to GRCh37, hg 19) (Figure 2B, D). Patient I final karyotype was: 46,XX,del(2)(q32.1 ~ q32.3).arr 2q31.2q32.3(178,121,127-194,943,948)x1 dn.

In patient II the deletion involves 37 genes from position 186,138,564 bp (clone A_16_P15961767) to 193,143,843 bp (clone A_18_P13572014) with the proximal breakpoint between 186,075,664 bp and 186,138,564 bp and the distal breakpoint between 193,143,843 bp and 193,194,619 bp (mapped according to GRCh37, hg 19) (Figure 2C, E). Oligoarray-CGH analysis also disclosed an unexpected 20p12.2p12.3 deletion in patient II spanning about 4 Mb and involving 13 genes from position 7,993,915 bp (clone A_16_P21064860) to position 11,937,684 bp (clone A_16_P03485400). The distal breakpoint is between 7,982,406 bp and 7,993,915 bp and the proximal breakpoint between 11,937,684 bp and 11,982,565 bp (mapped according to GRCh37, hg 19) (Figure 2F-H). Revisiting standard G-banded metaphases it is not possible to detect the 20p12.2p12.3 deletion with certainty, since it is within a cytogenetic difficult region (Figure 1D-E). MLPA SALSA P297-B1 for microdeletion syndromes (MRC-Holland) contains two probes for gene *PAK7,* included in the 20p deletion. It allowed us to confirm the proband’s deletion and to conclude it is de *novo* (paternity and maternity confirmed). Patient II final karyotype was: 46,XX,del(2)(q32.1 ~ q32.3).arr2q32.1q32.3(186,138,564-193,143,843)x1,20p12.3p12.2**(**7,993,915-11,937,684)x1 dn

## Discussion

Conventional cytogenetic evaluation of the reported patients revealed chromosome 2q32 deletions that were confirmed and refined by oligoarray-CGH. This molecular technique also revealed a 20p12 deletion in the Alagille syndrome region in patient II, enabling us to conclude that one of the reported patients presents two *de novo* deletions in two different chromosomes, both associated with described syndromes.

**Figure 3:**
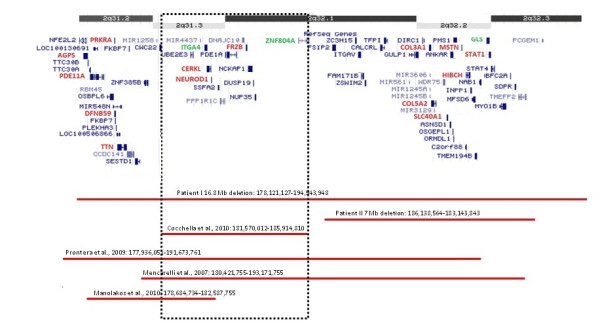
**Patients I and II chromosome 2 deletion compared to four previously reported patients.** OMIM Morbid Map genes are in red and other genes discussed in the text are in green. The dotted black rectangle delimitates the common deleted region among the patients. Breakpoints mapped according to GRCh37, hg 19.

In patient I, chromosome 2 deleted region involves 72 genes, with 14 reported in OMIM Morbid Map, and in patient II it involves 37 genes, all in common with patient I. Of the 37 genes that are deleted in both patients, 6 are described in the OMIM Morbid Map (Figure 3). One of those genes is *MSTN* (myostatin - OMIM 601788) whose loss-of-function mutations are associated with muscle hypertrophy, the increase of the size of muscle cells [7]. While reported patient I presents a muscular phenotype and an inadequate strength to her age, patient II does not present such features. We cannot disregard the reduced age of patient II, that might justify the actual absence of manifestations related to *MSTN* deletion, but this is also in accordance with Prontera *et al*. [4] argument that the manifestation of a muscular phenotype can occur in patients with larger 2q31.2q32.3 deletions rather than in those with more distal deletions.

Considered as a possible candidate gene for the behavioral phenotype in patients with 2q32 deletions is *GLS* (glutaminase, phosphate-activated - OMIM 138280), that encodes the major enzyme yielding glutamate from glutamine, whose importance is derived from its probable implication in behavior disturbances in which glutamate acts as a neurotransmitter [8,9]. This gene is deleted in both patients, and while patient I presents behavioral problems, patient II has an apparently normal psychomotor development to date. Meanwhile, according to the haploinsufficiency scores defined by the study of Huang *et al.*[10]*, GLS* has a score of 69%, making it a gene more likely to be haplosufficient. Considering this data and the absence of manifestations in reported patient II, the behavioral phenotype in patient I might be caused by the deletion of *ZNF804A* gene (zinc finger protein 804A - OMIM 612282), whose haploinsufficiency score is 32%. This score together with the described involvement of zinc finger genes in mental retardation make *ZNF804A* a candidate gene for cognitive and behavioral disturbances, consistently with previous findings involving other zinc-finger genes in mental retardation [11-16].

In patient I deleted region not concomitant with patient II, 8 other genes are described in OMIM Morbid Map, being the most relevant probably *FRZB* (frizzled-related protein - OMIM 605083) and *CERKL* (ceramide kinase-like - OMIM 608381) (Figure 3). *FRZB* gene codes for a protein that might play a role in skeletal morphogenesis [17] and thus can be a candidate gene for the craniofacial and hand/foot abnormalities described in patients with this deletion, since they commonly present tapering fingers at the hands and sandal gap at the feet [3-5] as in reported patient I. Haploinsufficiency of *CERKL* in patient I might justify her cognitive impairment when compared to patient II, since it codes for ceramide kinases that convert ceramide into ceramide-1-phosphate, both key mediators of cellular apoptosis and survival [18]. One gene also deleted only in patient I, *ITG4A* (integrin alpha-4 - OMIM 192975)*,* has been reported to be associated with autism in two different studies [19,20]. Cocchella *et al*. [5] postulated it to be a candidate gene for the behavioral manifestations and for the speech disorders observed in patients with 2q31.2q32.3 deletion, and the patient reported by Manolakos *et al*. [21], whose deletion includes *ITG4A,* also presents a speech limited to simple vocalization with lack of meaning. However, although our reported patient I presents behavioral problems she has an active speech, so we consider that absence of speech should be excluded of the core phenotype of 2q31.2q32.3 deletion as proposed by Cocchella *et al*. [5].

Concerning patient II, the cryptic imbalance detected by oligoarray-CGH at 20p12 is compatible with her clinical manifestations. Of the 13 genes deleted, 3 are described in OMIM Morbid Map, *MKKS* (McKusick-Kaufman syndrome - OMIM 604896), *PLCB1* (phospholipase C, beta 1 - OMIM 607120) and *JAG1* genes. *MKKS* and *PLCB1*genes are associated with McKusick-Kaufman syndrome (OMIM 236700) and Early Infantile Epileptic Encephalopathy-12 (OMIM 613722), respectively, both autosomal recessive disorders.

As already mentioned, *JAG1* gene haploinsufficiency is responsible for Alagille syndrome, whose clinical manifestations are highly variable [6]. Of the five clinical criteria for the diagnosis of AGS, patient II presents only manifestations at the cardiac level. This case might be one of those cases with low penetrance and expressivity [22] or the absence of additional clinical manifestations might be due to the reduced age of the proband. A challenge is put upon the clinical geneticist to do the follow-up of the child development, in order to establish a definitive phenotype and a correlation with the two reported chromosomal imbalances. The occurrence of both abnormalities would be expected to cause a more severe phenotype compared to the individual phenotype associated with each imbalance, which is not the case. We cannot rule incomplete penetrance of this imbalance. Taking into consideration that reported patient II does not present clinical features usually reported in 2q31.2q32.3 deletion syndrome, we can suggest that the critical region associated with the phenotype is outside patient II deleted region. As patient I manifests phenotypic features common to the other reported patients in the literature and their deletions overlap a common region, our results support Cocchella *et al*. [5] suggestion that the main phenotype is probably caused by the involvement of a small segment between 2q31.2q32.3. Furthermore we suggest that the critical region should involve bands 2q31.3q32.1 between 181 to 185 Mb, containing *CERKLFRZBZNF804A* and *ITG4A* genes discussed above (Figure 3). We did not consider the distal breakpoint of the patient reported by Manolakos *et al*. [21] since her phenotype is restricted to mental retardation and limited speech, probably related to the involvement of genes like *CERKL* and *ITG4A. FRZB* gene is normal in this patient, which is consistent with the absence of craniofacial and hand/foot abnormalities features that are common to patients with deletions involving this gene.

Establishing a syndrome with defined clinical characteristics is still difficult, due to the reported diversity of manifestations and phenotypes severity. Report of more cases fully characterized at a clinical and molecular level will be of major importance to definitely define the critical region of 2q31.2q32.3 deletion syndrome and to establish the core phenotype.

Patient II illustrates that multiple chromosome abnormalities in the same patient might be, to date, underestimated, because without oligoarray-CGH analysis and typical clinical manifestations of Alagille syndrome, the 20p12 deletion would not be identified and the proband’s phenotype would only be attributed to the 2q32 deletion. Array-CGH is a valuable and cost effective tool to detect cryptic genomic imbalances, to further characterize cytogenetically detected alterations and to accurately estimate the frequency of patients with multiple chromosome imbalances.

## Methods

### Conventional cytogenetic analysis

Conventional GTG banding, done according to standard protocols, was performed on peripheral blood lymphocytes of both probands and their progenitors [23]. Karyotypes were established according to the International System for Human Cytogenetic Nomenclature (ISCN) 2009 [24].

### Array-CGH

Precise determination of the breakpoints in the probands chromosomal imbalances was performed using Agilent SurePrint G3 Human Genome microarray 180 K (Agilent Technologies, Santa Clara, CA, USA), an oligonucleotide microarray containing approximately 180 000 60-mer probes with a 17 Kb average probe spacing. Genomic DNA of both patients was extracted from peripheral blood lymphocytes using Jetquick blood and cell culture DNA Midi Spin kit (Genomed, Löhne, Germany). Genomic DNA of a normal female control was obtained from Promega (Madison, WI, USA). Labeling was performed using Agilent Genomic DNA enzymatic labeling kit (Agilent) according to the manufacturers’ instructions. After hybridization, the oligoarray-CGH slide was scanned on an Agilent scanner, processed with Feature Extraction software (v10.7) and results were analyzed using Agilent Genomic Workbench (v6.0) with the following settings: ADM2 as aberration algorithm, threshold of 6.0, moving average 2 Mb.

MLPA was used to confirm oligoarray-CGH result for chromosome 20 [25] in patient II with commercially available SALSA P297-B1 for Microdeletion syndromes (MRC-Holland, Amsterdam, Netherlands) and was performed according to the manufacturers’ instructions.

## Consent

Written informed consent was obtained from the patients progenitors. A copy of the written consent is available for review by the Editor-in-Chief of this journal.

## Competing interests

The authors declare that they have no competing interests.

## Authors’ Contributions

SIF - carried out array-CGH and MLPA analysis and drafted the manuscript. EM - carried out cytogenetic analyses and provided valuable support for the manuscript drafting. MV- participated in the clinical evaluation and provided valuable support for the manuscript drafting. JS - participated in the clinical evaluation and provided valuable support for the manuscript drafting. JBM - participated in the design of the study, in array-CGH analysis and critically revised the manuscript. IMC - conceived the study, participated in its design and coordination and critically revised the manuscript. All authors read and approved the final manuscript.
